# Clinics and Exhibits of the New Jersey State Dental Society

**Published:** 1889-02

**Authors:** 


### CLINICS AND EXHIBITS OF THE NEW JERSEY STATE SOCIETY MEETING HELD AT ASBURY PARK, JULY, 1888.

*Dr. E. C. Kirk*, Phila., successfully implanted a superior central incisor for Dr. Cornwall, without the use of cocaine.

*Dr. T. S. Waters*, of Baltimore, built up with gold a superior first bicuspid, the palatine and anterior proximal sides of which were destroyed by decay. The electric mallet was used with six dry battery cells.

*Dr. Charles F. Wheeler*, of Albany, N. Y., exhibited his method of using gold with platinum and iridium, building up a right lateral and facing the cutting edges with the above-mentioned metals. He also built up a second lower molar completely broken down to the margin of the gums, and faced the entire grinding surface with the same metal.

*Dr. William B. Finney*, of Baltimore, filled with gold a right superior first bicuspid, the distal surface and crown gone. The tooth was filled from the apex with the electric mallet, using a Detroit dental motor, consuming nearly three hours time and one-eighth ounce of foil.

*Dr. C. A. Timme*, of Hoboken, N. J., exhibited partial cases of pure gold deposited on the cast by electricity the thickness of twenty-four gauge plate. The plates are quite stiff, and can be soldered if necessary. The lower plates are stiffened by a gold wire soldered on the lingual surface. They are made for the profession by Durand & Co., of Newark, N. J.

*Dr. S. C. G. Watkins*, of Montclair, exhibited new shapes of amalgam instruments.

*Dr. G. L. Curtis*, of Syracuse, implanted a second inferior molar for Dr. L. N. Seymour of Asbury Park. While the operation was necessarily difficult, owing to the location and the recession of the aveolar walls, it was eminently successful. The patient being extremely sensitive, a twenty-five per cent. solution of cocaine was employed locally with good results. The operation occupied about thirty minutes. The tooth was secured to the third molar by means of a palatinum wire staple to prevent movement of the ingrafted organ until union was complete. The tooth was examined two days later and was doing well, the patient having suffered no special inconvenience.

### EXHIBITS.

The S. S. White Dental Manufacturing Company had on exhibition dental chairs, electric motor, dental engine, revolving fans, oxygen inhaler, new implantation instruments, crown instruments, matrices, and a full line of dental instruments generally.

Gideon Sibley, of Philadelphia, porcelain teeth, dentifrices, Sibley's gold foil, dental instruments, etc.

Seabury & Johnson, of New York, antiseptic cotton, hydronapthol, lints, and disinfectants.

A. W. See & Co., of New York, gold foils, amalgams, dental engines, operating chair, etc.

Dr. Ivory, of Philadelphia, rubber damp clamp.

Mr. Green, of Camden, instruments and dental engine clamps.

The Florence Manufacturing Company, of New York, tooth brushes.

Welch & Co., of Philadelphia, porcelain teeth, dental equipments, etc.

Ward's Electro Metallic Dental Plate was exhibited by the inventor and Dr. E. E. Clarke, of Newark. Gold is deposited on a silver plate for use in artificial dentures.

### COMMITTEE REPORTS.

On motion of Dr. Watkins, a vote of thanks was tendered to all the gentlemen who had read papers at the meeting or operated in the clinics.

On motion of Dr. Levy, a special vote of thanks to Dr. J. L. Smith for kindly lending his magic lantern, and giving his services in its use.

A special committee of three, consisting of Drs. Levy, Brown and Meeker were appointed to wait upon the proprietor of the West End Hotel and tender the thanks of the Association for kind attentions to the Society.

The Committee appointed on the President's Address would most respectfully recommend the adoption of the following Preamble and Resolutions:

*Whereas*, It has pleased the All-wise Ruler of the Universe, in His inscrutable wisdom, to remove from our midst our late member and companion, John W. Scarborough, while in the very vigor of his manhood and zenith of his usefulness—a man of honesty, integrity, and conscientiousness in the performance of his duty as a professional man, and citizen, and true to all the obligations of life, both morally and socially—

*Resolved*, That while we bow to the decree of the Most High, we will place on record our appreciation of his services as a member of our Society, and acknowledge his faithfulness in his attendance on its many meetings, and his promptness in fulfilling the duties imposed upon him.

*Resolved*, That a copy of this Preamble and Resolutions be recorded in the minutes of our Society.

They would also recommend, that in case of death occurring to any of our members, that the Chairman of the Executive Committee, on being notified of the fact, shall appoint a committee to attend the funeral of the deceased member, and report at the next meeting of the Society.

[Signed.]

FRED A. LEVY,<br>J. HAYHURST,<br>S. C. G. WATKINS, } *Committee.*

Adjourned, *sine die.*

